# Unraveling Individual Differences In The HIV-1 Transgenic Rat: Therapeutic Efficacy Of Methylphenidate

**DOI:** 10.1038/s41598-017-18300-2

**Published:** 2018-01-09

**Authors:** Kristen A. McLaurin, Hailong Li, Rosemarie M. Booze, Amanda J. Fairchild, Charles F. Mactutus

**Affiliations:** Program in Behavioral Neuroscience, Department of Psychology, University of South Carolina, Columbia, SC 29208 USA

## Abstract

Despite the heterogeneity of HIV-1 associated neurocognitive disorders (HAND), assignment of categorical diagnoses based on the level of impairment (e.g., Frascati criteria) obfuscates the well-acknowledged variability observed within the population of HIV-1+ individuals. The present study sought to elucidate the natural heterogeneity in adult HIV-1 transgenic (Tg) rats using three interrelated aims. First, heterogeneity of the HIV-1 transgene was examined using a pretest-posttest design to assess therapeutic efficacy of oral self-administration (OSA) of methylphenidate (MPH; 2.4 ± 0.2 mg/kg), targeting neurotransmitter alterations in HIV-1, on temporal processing. Approximately 42% of HIV-1 Tg animals displayed an improvement in temporal processing following OSA of MPH. Second, repeated OSA of MPH (22–27 days) altered dendritic spine morphology in layer II-III pyramidal neurons in the medial prefrontal cortex. HIV-1 Tg animals exhibited a population shift towards longer spines with decreased head diameter on lower order branches; a shift associated with temporal processing impairment. Third, in HIV-1 Tg animals, dendritic spine backbone length (µm) was associated with temporal processing impairment; a brain/behavior relationship not observed in control animals. Assessing the therapeutic efficacy of MPH revealed heterogeneity in the neural mechanisms underlying neurocognitive impairments, providing a key target for individualized therapeutic and diagnostic approaches for HAND.

## Introduction

HIV-1 associated neurocognitive disorders (HAND) persist, despite treatment with combination antiretroviral therapy (cART). In the post-cART era, approximately 40–70% of HIV-1 seropositive individuals are afflicted with HAND [e.g.^1,2^], characterized by deficits in attention, memory, and executive function^2,3^. Although the neuropsychological profile of HAND is naturally heterogeneous, categorical diagnoses are commonly employed [e.g.^4^], obfuscating the well-acknowledged variability observed within the population of HIV-1 seropositive individuals^5^. Understanding the heterogeneity of neurocognitive impairments (NCI) in the HIV-1 transgenic (Tg) rat, may help unravel therapeutic strategies and diagnostic screening tools for more individualized treatment of HAND.

Given the continued prevalence of HAND in the post-cART era, there is a critical need to develop adjunctive therapeutics for HIV-1 seropositive adults. Alterations in dopamine (DA) system dysfunction, which have been implicated as a potential neural mechanism for the NCI observed in HAND, may provide a key target for the development of therapeutic treatments. Clinical studies in the post-cART era have revealed greater brain atrophy in areas rich in DA, including the basal ganglia^6^, substantia nigra^7^ and caudate nucleus^6^. Decreases in DA in the substantia nigra^7^ have been associated with NCI. Preclinical assessments of other prominent NCI in HIV-1, including deficits in temporal processing [e.g.^8,9^], habituation [e.g.^9,10^], and sustained attention [e.g.^11^], provide additional evidence for DA system dysfunction.

The underlying mechanisms of DA system dysfunction in HIV-1 have also been explored. Specifically, DA system dysfunction in HIV-1 may result from impairment in the dopamine transporter (DAT), which is targeted by HIV-1 viral proteins [e.g.^12^] and critical for cognitive function^13^. Positron emission tomography (PET) scans revealed decreased DAT levels in HIV-1 seropositive individuals^14,15^, manifesting in deficits which were related to neurocognitive performance^15^. Additionally, preclinical studies in the HIV-1 Tg rat revealed neurochemical changes, including decreased DAT mRNA^16^.

Norepinephrine (NE), another neurotransmitter involved in cognitive functioning in the prefrontal cortex [PFC;^17^], may also be involved in the pathogenesis of HIV-1. Both *in vitro* and *in vivo* studies have demonstrated the role of NE on viral replication and impaired response to highly active antiretroviral therapy [HAART; e.g.^18,19^]. In a sample of HIV-1 seropositive individuals, higher baseline levels of NE were predictive of disease progression [i.e., greater loss of CD4 cells and increased viral load;^20^]. Other studies have also reported the relationship between CD4 cell count and NCI, suggesting that a decreased CD4 cell count was associated with an increased odds of developing NCI^21^. Thus, NE may play a direct and/or indirect role on the development of NCI via its relationship with viral progression; a role which, to date, has not been systematically evaluated.

The therapeutic benefits observed following administration of methylphenidate (MPH) may be due to the blockade of the DAT^22–25^ and/or the norepinephrine transporter [NET;^24,25^]. MPH, a psychostimulant medication, has been widely used to enhance cognitive functions in multiple neurocognitive disorders, including attention-deficit hyperactivity (ADHD) disorder [e.g.^26^], apathy [e.g.^27^], multiple sclerosis [e.g.^28^], and traumatic brain injury [e.g.^29^]. However, significant individual variability in therapeutic efficacy of MPH has been observed, with approximately 10–30% of ADHD patients failing to adequately respond to treatment^30^. The potential therapeutic utility of MPH as a treatment for NCI in HIV-1 has also been suggested^31,32^. Sustained-release MPH treatment enhanced cognitive improvements, relative to a placebo control, in opiate-dependent HIV-1 seropositive individuals^31^. A subsequent study suggested that HIV-1 seropositive individuals with a greater degree of cognitive slowing may be most responsive to MPH treatment; a potential constraint on the therapeutic efficacy of MPH for HAND^32^. Thus, the present study examined the therapeutic efficacy of MPH for the treatment of HAND as a vehicle to elucidate the natural heterogeneity in HIV-1 Tg rats.

Although multiple diagnostic criteria have been developed for the assessment of HAND in the post-cART era, including clinical ratings [e.g.^33^], deficits scores [e.g.^34^], and HAND diagnostic classifications/Frascati criteria^4^, they have several limitations. Specifically, all of the methods recommend a complete battery of neuropsychological testing, which requires use of specialized equipment, long administration times, neuropsychological expertise, and appropriate normative data; luxuries that may not be available in low-resource countries, which carry the largest burden of HIV^35^. Additionally, use of the Frascati criteria to assess neurocognitive impairment in a group of HIV-1 infected and –uninfected children revealed a high “false-positive” rate^36^. Most critically, however, regardless of the diagnostic criteria employed, patients are ultimately categorically classified^37^. Thus, the development of an accurate and reliable diagnostic screening tool, without categorical classification, for the detection of HAND in the post-cART era is of great clinical significance^35^.

Temporal processing, a potential elemental dimension of HAND, may provide an innovative diagnostic screening tool for HAND. Prepulse inhibition (PPI) of the auditory startle response (ASR), gap prepulse inhibition (gap-PPI), and gap threshold detection have been promoted as translational experimental paradigms for the examination of temporal processing^38–40^. Specifically, gap threshold detection, of interest in the present study, relies on two stimuli: a punctate prestimulus (e.g., a gap in background noise) and a startle stimulus. The length of gap duration is manipulated to directly assess temporal processing. A prominent monotonic relationship is observed in gap threshold detection [i.e., a linear decrease in ASR as gap duration increases;^40^], providing a distinct advantage for clinical diagnosis and assessment. A previous examination of the potential utility of auditory gap threshold detection revealed that two measures (i.e., slope of the auditory gap threshold detection curve and ASR amplitude for gap threshold detection at 5 msec) identified the presence of the HIV-1 transgene with 91.1% accuracy^41^.

Thus, given the need for adjunctive therapeutic treatments and novel diagnostic screening tools, the present study sought to elucidate the natural heterogeneity in adult HIV-1 Tg rats, without categorical classification, using three interrelated aims. First, heterogeneity of the HIV-1 transgene was examined using a pretest-posttest experimental design assessing the therapeutic efficacy of oral self-administration (OSA) of MPH on temporal processing. Second, to examine the effect of repeated (22–27 days) OSA of MPH on dendritic spine morphology in layer II-III pyramidal neurons, targets of DA and NE afferents, of the medial prefrontal cortex (mPFC). Third, to investigate the heterogeneity in the HIV-1 transgene by examining the effect of categorical classification schemes on the diagnostic utility of gap threshold detection and presence of a brain/behavior relationship between temporal processing and dendritic spine morphology. Unraveling the natural heterogeneity in the HIV-1 transgene may provide key targets for individualized therapeutic and diagnostic approaches for the treatment of HAND.

## Methods

### Animals

The natural heterogeneity in the HIV-1 transgene was assessed in ovariectomized female Fischer (F344/N; Harlan Laboratories Inc., Indianapolis, IN) rats (HIV-1 Tg, *n* = 19; control, *n* = 20) by examining the potential therapeutic efficacy of MPH. Given the prevalence of HIV-1 seropositive women worldwide, accounting for approximately 51% of all HIV-1 seropositive individuals^42^, and the underrepresentation of the female sex in clinical and preclinical assessments of MPH^43^, female animals were evaluated in the present study. All animals were pair- or group-housed throughout the duration of the experiment. Rodent food (2020X Teklad Global Extruded Rodent Diet (Soy Protein-Free)) and water were available *ad libitum* with the exception of water restriction during preliminary training.

Guidelines established by the National Institutes of Health (NIH) were used to house animals in AAALAC-accredited facilities. The targeted environmental conditions for the animal facility were 21° ± 2 °C, 50% ± 10% relative humidity and a 12-h light:12-h dark cycle with lights on at 0700 h (EST). The University of South Carolina Institutional Animal Care and Use Committee (IACUC) approved the project under federal assurance (# A3049-01). All animal procedures were performed in accordance with this federal assurance.

### Experimental Timeline

**Figure 1:**
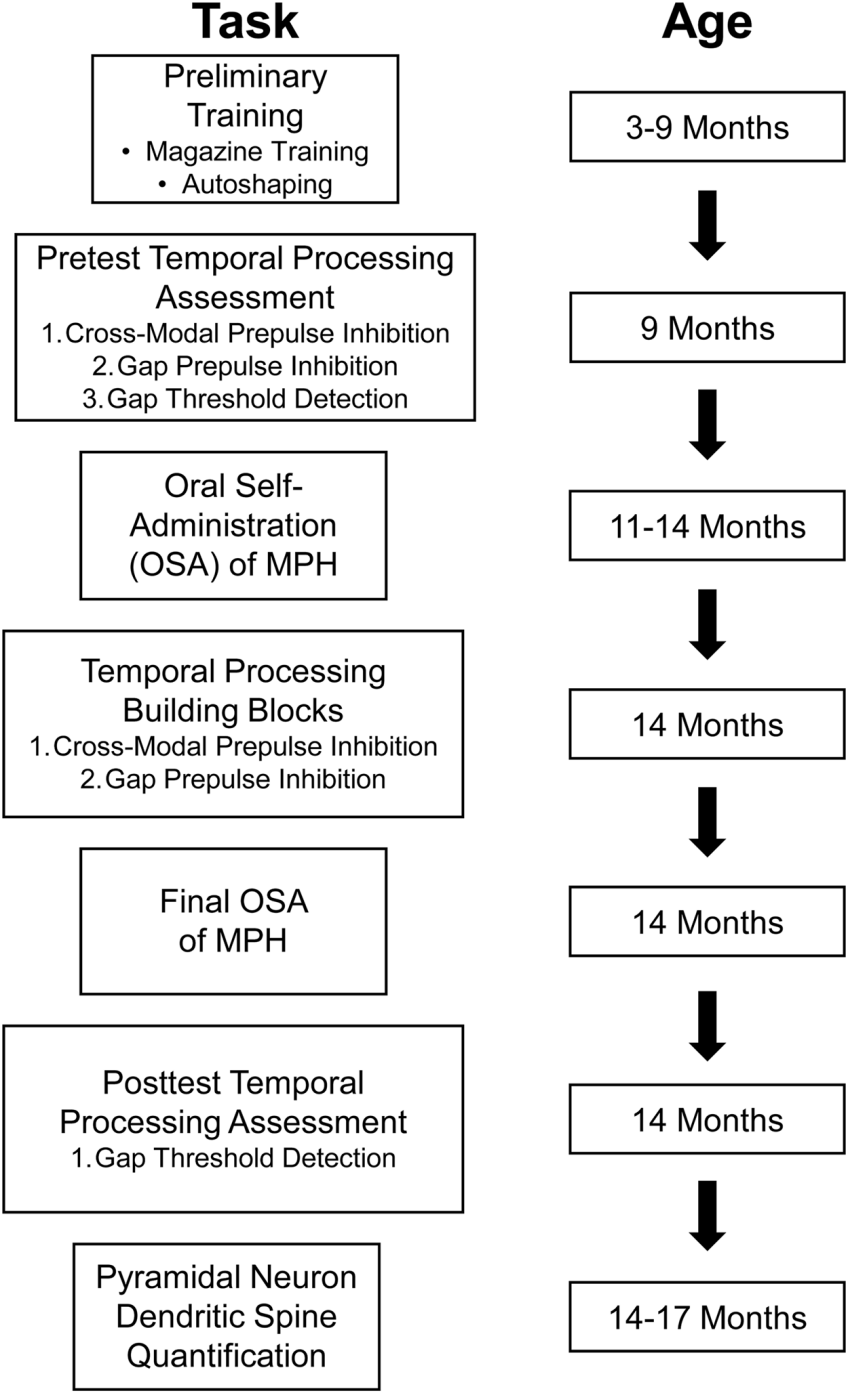
Experimental Timeline.

The experimental timeline for operant conditioning, pretest temporal processing assessments, OSA of MPH, posttest temporal processing assessments, and pyramidal neuron dendritic spine quantification is illustrated in Fig. 1.

### Operant Conditioning

#### Apparatus

Behavioral training and testing was conducted in 18 operant chambers (ENV-008; Med-Associates, St. Albans, VT, USA) located in sound-attenuating enclosures. One wall of each chamber consisted of two retractable metal levers (ENV-112BM), a recessed food dipper (ENV-202M) between the two levers, and a 28-V white cue light located above each response lever. Following the completion of the relevant response requirement, a dipper equipped with a 30 μl cup attached to the end of the dipper arm was raised into the food receptacle, allowing access to sucrose or MPH. The cue light, which was 3 cm in diameter, was used to signal time-outs. Head entries into the food receptacle were detected using an infrared sensor (ENV-254-CB). Med-PC computer interface software was used to control signal presentation, lever operation, reinforcement delivery, and data collection.

#### Drugs

Racemic MPH hydrochloride (Sigma-Aldrich Corporation, St. Louis, MO; 50% I.d active stereoisomers) was prepared prior to administration by dissolving MPH with sucrose (5–26%) in double distilled water (ddH_2_O).

#### Preliminary Training

A standard operant conditioning training paradigm (i.e., magazine training, autoshaping), was used to train animals according to previous research^44^. Animals were trained to lever press using 5% (w/v) sucrose using a fixed ratio 1 (FR-1) reinforcement schedule. All animals learned the operant response prior to oral self-administration of MPH.

#### OSA Dosing of MPH

At approximately eleven months of age, animals were trained to orally self-administer MPH on a daily alternating schedule (i.e., MPH, sucrose, MPH, sucrose) during a 42 minute test session. Both the right and left levers were presented in the operant chamber. An FR1 training schedule was used, such that animals were reinforced with either MPH or sucrose after each response on the active levers (i.e., either left or right). After the completion of each operant session, the actual dose administered by the animals was calculated using the following formula: [(Number of Rewards × 30 µl × Concentration of MPH × 1000)/(1000 × Animal’s Body Weight)], where 30 µl represented the size of the dipper. The targeted dose was 4 mg/kg when an animal acquired 120 rewards within the 42 minute test session (i.e., time, not rewards, indicated the termination of the session). During acquisition training, through stable performance, HIV-1 Tg and control animals self-administered comparable doses of MPH (Mean ± Standard Error), independent of sucrose concentration [0%: Control (1.7 ± 0.1 mg/kg), HIV-1 Tg (1.5 ± 0.1 mg/kg); 5%: Control (2.0 ± 0.1 mg/kg), HIV-1 Tg (2.0 ± 0.1 mg/kg); 10% or 26%: Control (3.7 ± 0.4 mg/kg), HIV-1 Tg (3.5 ± 0.4 mg/kg)].

OSA of MPH was maintained by allowing animals to OSA MPH (dissolved in 10% or 26% sucrose) using a 42 minute test session, with a maximum of 120 responses (i.e., both the time and number of rewards indicated the termination of the session). The final OSA of MPH dosing immediately preceded the posttest assessment of auditory gap threshold detection. At the final dosing, HIV-1 Tg and control animals self-administered doses of MPH that were not statistically different [HIV-1 Tg: 2.1 ± 0.3 mg/kg; Control: 2.6 ± 0.2 mg/kg; *t*(31.7) = 1.6 *p* > 0.05]. In total, animals orally self-administered MPH for between 22–27 days.

### Temporal Processing Assessments

#### Apparatus

A 10 cm-thick double walled isolation cabinet (external dimensions: 81 × 81 × 116-cm) (Industrial Acoustic Company, Inc., Bronx, NY), offering 30 dB(A) of sound attenuation, enclosed the startle platform (SR-Lab Startle Reflex System, San Diego Instruments, Inc., San Diego, CA) instead of the 1.9 cm thick ABS plastic or laminate cabinets typically offered with this system. The ambient sound level in the chamber without any stimuli was 22 dB(A). Auditory prepulse and stimuli (frequency range 5k-16k Hz) were delivered using a high-frequency loudspeaker of the SR-Lab system (model #40-1278B, Radio Shack, Fort Worth, TX), affixed 30 cm above the Plexiglas. Measurement and calibration of sound levels were conducted using a sound level meter (model #2203, Bruel & Kjaer, Norcross, GA) with the microphone placed inside the Plexiglas cylinder. The test cylinder was deflected by the animal’s response to the startle stimulus; a piezoelectric accelerometer, attached to the bottom of the cylinder, converted the responses into analog signals, which were digitized (12 bit A to D) and saved to a hard disk. Response sensitivities were calibrated using a SR-LAB Startle Calibration System.

#### Auditory Gap Threshold Detection

Auditory gap threshold detection was administered using a 20-min test session, beginning with a 5-min acclimation period in the dark. Background white noise (70 db(A)) was presented throughout the acclimation period. Following the acclimation period, 6 pulse-only ASR trials were presented with a fixed intertrial interval (ITI; 10-sec). Thirty-six trials were presented at the 50-msec ISI, with gap durations presented in 6-trial blocks interdigitated using a Latin-square experimental design. Trials employed gap durations of 5, 10, 20, 30, 40, and 50 msec and had a variable ITI from 15–25-sec. Analyses were conducted on the mean peak ASR amplitude values.

#### Procedure

Cross-modal PPI and auditory gap-PPI, critical building blocks for auditory gap threshold detection^45^, were conducted prior to both pretest and posttest auditory gap threshold detection assessments. Pretest temporal processing assessments were conducted at approximately 9 months of age, while posttest assessments occurred at approximately 14 months of age. Immediately following the completion of the final OSA of MPH test session (i.e., when the test session timed out after 42-minutes or an animal received the maximum of 120 rewards), animals were assessed in auditory gap threshold detection.

### Pyramidal Neuron Dendritic Spine Quantification

#### Preparation of Tissue

Animals were sacrificed between 14 and 17 months of age following repeated OSA of MPH. Animals were deeply anesthetized using sevoflurane (Abbot Laboratories, North Chicago, IL) and transcardially perfused using methodology adapted from Roscoe *et al*.^46^. Brains were dissected and post-fixed in 4% paraformaldehyde for 10 minutes. A rat brain matrix (ASI Instruments, Warren, MI) was used to cut 1 mm thick coronal slices, which were washed 3x in PBS, notched for orientation, and placed in tissue cell culture plates (24 well plate; Corning, Tewksbury, MA).

#### DiOlistic Labeling

**Figure 2:**
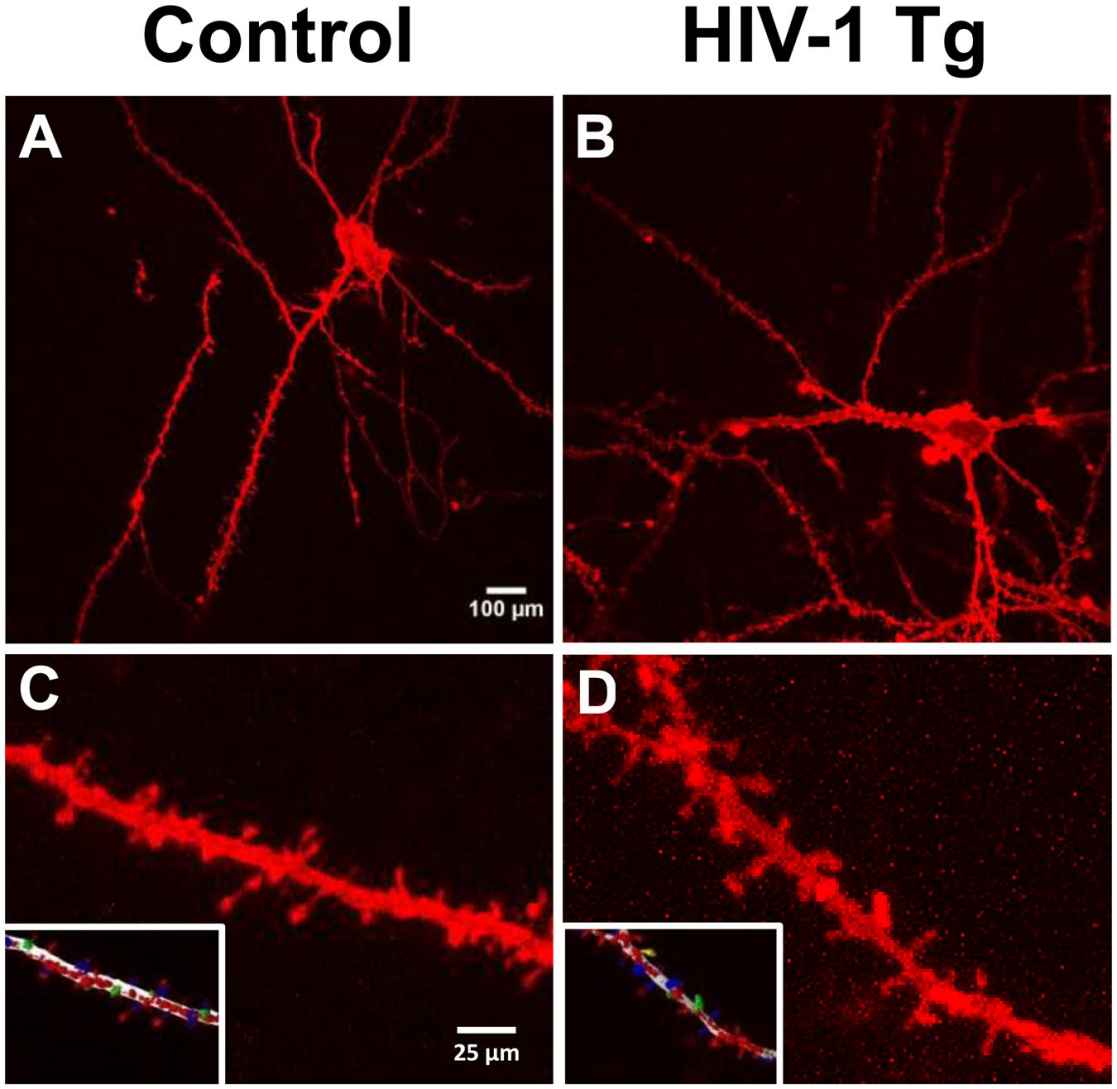
DiOlistic labeling of pyramidal neurons in layers II-III medial prefrontal cortex (mPFC). Pyramidal neurons in both control (**A**) and HIV-1 Tg (**B**) were characterized by one large apical dendrite and several smaller basal dendrites^55^. HIV-1 Tg animals (**D**) exhibited a population shift towards longer dendritic spines with decreased head diameter relative to control animals (**C**). The inset shows the tracing and classification of dendritic spines on Neurolucida 360.

In the present study, DiOlistic labeling (Fig. 2) was completed using the technique described by Seabold *et al*.^47^. DiOlistic labeling introduces indocarbocynanine dye via ballistic methods providing an opportunity to visualize cells, including neurons that are traditionally difficult to examine^47^. Relative to more traditional methods (i.e., Golgi-Cox silver impregnataion, microinjection), DiOlistic labeling provides multiple advantages. Specifically, DiOlistic labeling results in minimal tissue damage, distinguishes morphological characteristics in spines, and does not disrupt cellular processes^48,49^. The ability of DiOlistic labeling to distinguish morphological characteristics in spines, including dendritic spine shape and branching pattern^47^, is of particular relevance to the present study.

Methodology for the preparation of DiOlisitic cartridges, preparation of Tefzel tubing, and DiOlistic labeling was adapted from Roscoe *et al*.^46^. In brief, DiOlistic cartridges were prepared using 170 mg of tungsten beads (Bio-Rad, Hercules, CA) dissolved in 99.5% pure methylene chloride (Sigma-Aldrich, St. Louis, MO). 100 µl of the bead solution was placed on a glass slide and 150 µl of crystallized DiI (6 mg; Invitrogen, Carlsbad, CA), dissolved in methylene chloride, was titrated on top. The dye/bead mixture was allowed to air dry, collected with wax-coated weigh paper, and transferred to a 15 ml conical tube (BD Falcon, San Jose, CA) with 3 ml ddH_2_O and sonicated for 10–15 minutes. The dye/bead mixture was slowly drawn into Tefzel tubing (IDEX Health Sciences, Oak Harbo, WA) and placed in the tubing prep station (Bio-Rad) for 5 minutes. Tubing was allowed to fully dry under nitrogen gas and was cut into 13 mm segments.

DiOlistic labeling was completed using the Helios gene gun (Bio-Rad, Hercules, CA), which was loaded with previously prepared DiOlistic cartridges. Ballistic delivery was conducted with Helium gas flow adjusted to 80 PSI and particles delivered through 3 µm pore filter paper. The barrel of the Helios gene gun was placed approximately 2.5 cm away from the sample. Following DiOlistic labeling, sections were washed 3x in PBS and stored overnight at 4 °C to allow dye diffusion. Pro-Long Gold Antifade (Invitrogen, Carlsbad, CA) was used to mount tissue sections, which were cover slipped (#1 cover slip; Thermo Fisher Scientific, Waltham, MA) and stored in the dark at 4 °C.

#### Pyramidal Neuron Dendritic Analysis and Spine Quantification

Pyramidal neurons from layers II-III of the mPFC, located approximately 3.7 mm to 2.2 mm anterior to Bregma^50^, were analyzed. For dendritic spine analysis, Z-stack images were obtained on three to four pyramidal neurons from each animal [HIV-1 Tg (*n* = 14), F344/N Control (*n* = 14)] using a Nikon TE-2000E confocal microscope utilizing Nikon’s EZ-C1 software (version 3.81b). Images were obtained using the methodology previously reported [i.e., 60x, Z-plane intervals of 0.15 µm; helium-neon laser;^46^].

Analysis of spine parameters was conducted using Neurolucida 360, utilizing the AutoNeuron and AutoSpine extension modules (MicroBrightfield, Williston, VT). Prior to the analysis of spine parameters, neurons were blinded to prevent experimenter bias. One neuron from each animal was chosen for analysis of spine parameters based on selection criteria (i.e., continuous dendritic staining, low background/dye clusters).

#### Spine Parameters

Dendritic spine morphology was analyzed using backbone length (µm), head diameter (µm), and volume (µm^3^). Spine parameters were defined using well-accepted previously published results, with a goal of representing at least 80% of the total number of dendritic spines to account for the natural heterogeneity. Dendritic spine backbone length was defined as measures between 0.1 to 4.0 µm^51^. Dendritic spine head diameter was defined as measures between 0 to 0.8 µm^52^. Dendritic spine volume was defined as measures between 0.05 to 0.8 µm^3^ ^53^. Dendritic spines were automatically classified (i.e., thin, mushroom, stubby, filopodia) in Neurolucida 360.

### Statistical Analysis

Statistical analyses were conducted using SAS 9.4 (SAS/STAT Software 9.4, SAS Institute, Inc., Cary, NC). Figures and regression analyses were completed using GraphPad Prism 5 (GraphPad Software, Inc., La Jolla, CA). Three-dimensional surface plots were created using MatLab R2017a (MathWorks, Natick, MA). A discriminant function analysis (DFA), to examine the diagnostic utility of gap threshold detection, was conducted using SPSS Statistics 24 (IBM Corp., Somers, NY). All statistical tests were evaluated against a *p* ≤ 0.05 alpha criterion.

Genotype (i.e., HIV-1 Tg vs Control) differences were examined to assess the effect of MPH on temporal processing and dendritic spine morphology in the population of animals sampled. Data for auditory gap threshold detection were log transformed, with a goal of relating stimulus intensity (i.e., duration) to sensation magnitude according to Steven’s Power Law^54^. Auditory gap threshold detection was analyzed using a mixed-design analysis of variance (ANOVA) and restricted maximum likelihood estimation of model parameters. Test session (pretest vs. posttest), gap duration, and trial served as the within-subjects factors, while genotype (HIV-1 Tg vs. Control) served as the between-subjects factor.

Dendritic spine morphology, including backbone length, head diameter, and volume, was analyzed using Pearson’s chi-squared (χ^2^) test of independence to examine the effect of genotype (HIV-1 Tg vs. Control) on the distribution of dendritic spine morphology. The number of dendritic spines in each bin was used for the χ^2^ analysis. Given the preponderance of thin spines in both HIV-1 Tg and control animals, a χ^2^ was used to assess the distribution of spines on each branch.

Given the natural heterogeneity in HIV-1 Tg animals, subsequent analyses were conducted to examine individual differences in the therapeutic efficacy of MPH in HIV-1 Tg animals. The slope for each animal’s unique pretest and posttest auditory gap threshold detection curve was calculated using GraphPad Prism 5. A change score, calculated as ((Pretest Slope of the Auditory Gap Threshold Detection Curve)-(Posttest Slope of the Auditory Gap Threshold Detection Curve)), was used to assess the magnitude and valence of impairment following OSA of MPH. The more positive the change score, the greater the improvement in temporal processing at the posttest assessment relative to the pretest assessment. Conversely, the more negative a change score, the greater impairment in temporal processing at the posttest assessment relative to the pretest assessment.

Dendritic spine morphology and branch order were subsequently analyzed for each animal to examine the effect of change score on the shift in dendritic spine morphology. For the statistical analysis, animals were binned into 20^th^ percentile groupings based on their change score. A Pearson’s χ^2^ test of independence was conducted to directly quantify the overall effect of change score on the shift in dendritic spine morphology.

The effect of arbitrary classification on the HIV-1 Tg rat was assessed using a DFA. At the pretest assessment, one variable (i.e., gap threshold detection slope) was used to diagnose animals based on the presence of the HIV-1 transgene. At the posttest assessment, HIV-1 Tg animals were arbitrarily classified using the magnitude and valence of their change score into two groups: Positive Change Score (i.e., improvement in temporal processing) and Negative Change Score (i.e., temporal processing impairment). Following the arbitrary group classification scheme, one variable (i.e., gap threshold detection slope) was used to diagnose animals. A brain-behavior relationship, as a method to include the natural heterogeneity in HIV-1 Tg animals, between auditory gap threshold detection and dendritic spine morphology was examined using Pearson correlation and regression analyses. One HIV-1 Tg animal was removed from the brain/behavior relationship, including the statistical analysis, because it was a statistical outlier with an auditory gap threshold detection slope greater than 2.5 standard deviations away from the mean.

### Data availability

All relevant data are within the paper.

## Results

### Examination of Genotype Effects

#### OSA of MPH failed to ameliorate temporal processing deficits in the population of HIV-1 Tg animals sampled

**Figure 3:**
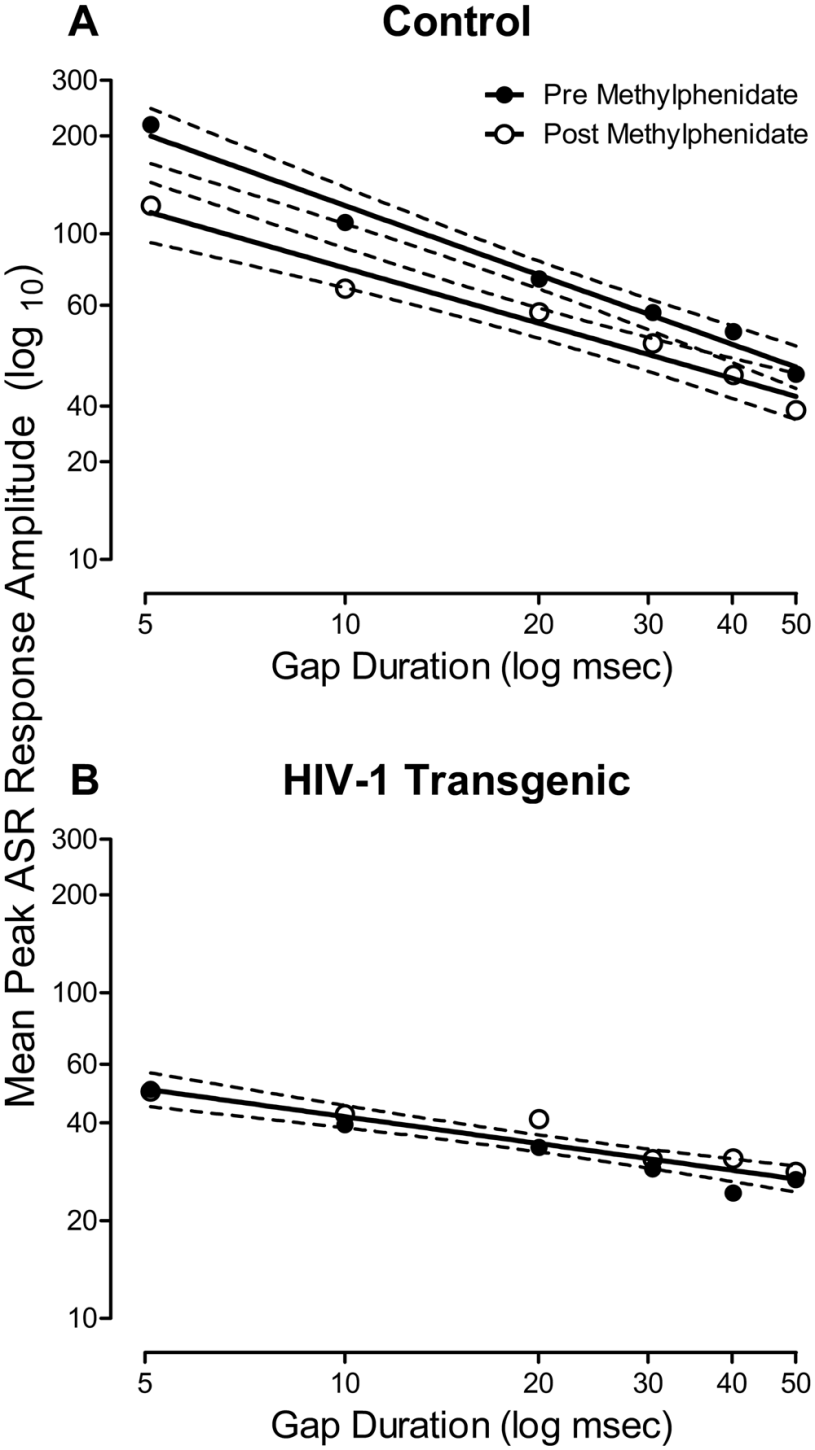
Mean peak ASR startle response (±95% Confidence Interval) is presented as a function of oral self-administration (OSA) of methylphenidate (MPH; pretest vs. posttest) for control (**A**) and HIV-1 Tg (**B**) animals. Regardless of genotype or methylphenidate exposure, a linear decrease in mean peak ASR amplitude was observed as gap duration increased. (**A**) In control animals, a relatively flatter auditory gap threshold detection curve was observed following the final day of OSA of MPH relative to the pretest assessment. (**B**) In HIV-1 Tg animals, OSA of MPH failed to ameliorate temporal processing deficits, evidenced by no change (i.e., a global fit) in the slope of the auditory gap threshold detection curve.

Examination of the population of HIV-1 Tg animals sampled revealed that OSA of MPH failed to ameliorate temporal processing deficits, assessed using auditory gap threshold detection (Fig. 3).

During the pretest assessment, a linear relationship between ASR and gap duration was observed in both HIV-1 Tg and control animals, best fit using a first-order polynomial [*R* ^2^s ≥ 0.95]. Significant differences in the well-described function were observed between groups [*F*(2, 8) = 199.0, *p* ≤ 0.001]. Specifically, HIV-1 Tg animals (β_1_ = −0.30 ± 0.086) displayed a significantly slower decrease in ASR relative to control animals (β_1_ = −0.71 ± 0.128), suggesting a relative insensitivity to the manipulation of gap duration.

Following the final dose of OSA of MPH, significant alterations in the first-order polynomial [*R* ^2^s ≥ 0.92], which provided a well-described fit, were observed in control [*F*(2, 8) = 19.6, *p* ≤ 0.001], but not HIV-1 Tg animals [*p* > 0.05]. Control animals exhibited a significant decrease in the slope of the auditory threshold detection function at the posttest (β_1_ = −0.56 ± 0.15 (95% CI) relative to the pretest assessment (β_1_ = −0.71 ± 0.128 (95% CI).

Results from the mixed-design ANOVA confirmed these observations, revealing a significant assessment × genotype interaction [*F*(1, 37) = 18.3, *p* ≤ 0.001] and a significant duration × genotype interaction [*F*(5, 185) = 12.7, *p* ≤ 0.001]. A significant main effect of assessment [*F*(1, 37) = 15.2, *p* ≤ 0.001], duration [*F*(5, 185) = 87.0, *p* ≤ 0.001] and genotype [*F*(1, 37) = 32.6, *p* ≤ 0.001] were also observed. Thus, OSA of MPH failed to ameliorate temporal processing deficits in the population of HIV-1 Tg animals sampled.

#### Repeated OSA of MPH altered the distribution of dendritic spine morphology in layers II-III pyramidal neurons of the medial prefrontal cortex

**Figure 4:**
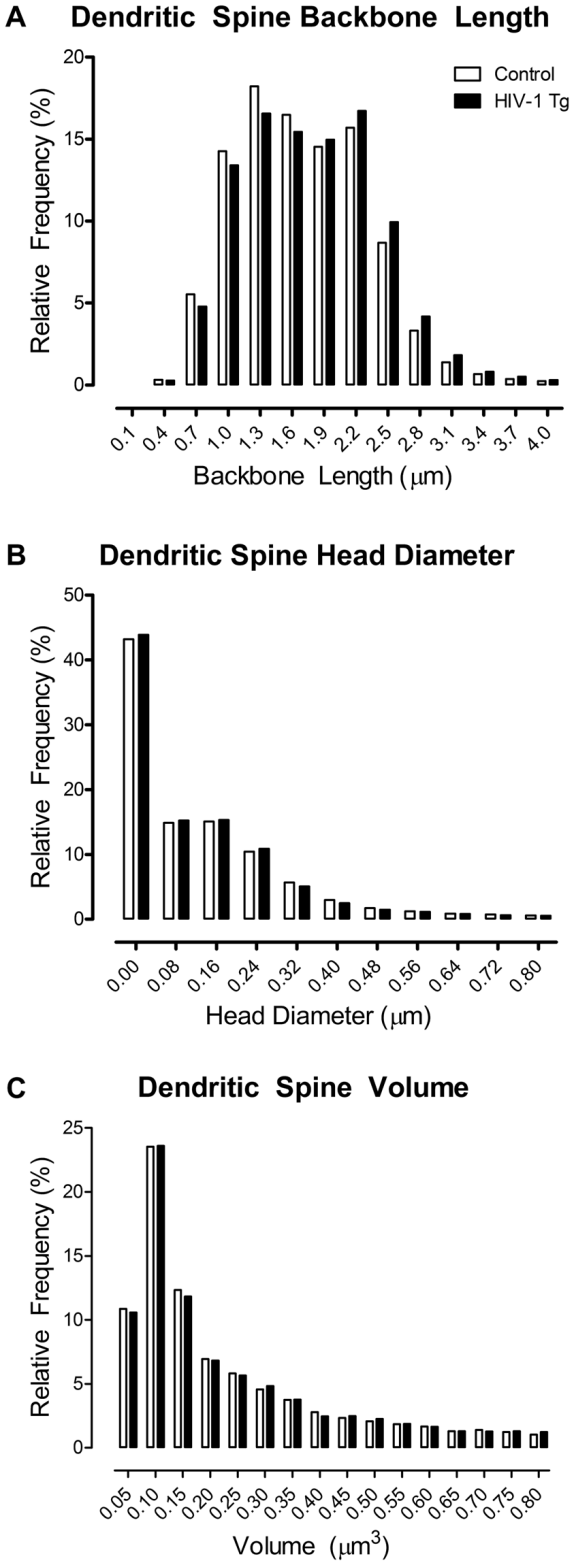
Histograms representing the distribution of dendritic spine backbone length (**A**), head diameter (**B**), and volume (**C**) following repeated oral self-administration (OSA) of methylphenidate (MPH) exposure are illustrated as a function of genotype (HIV-1 Tg vs. Control). HIV-1 Tg animals displayed a population shift towards longer dendritic spines (**A**) with decreased head diameter (**B**) relative to control animals. No significant differences in dendritic spine volume were observed between genotype (**C**).

Measurement of dendritic spine parameters, including backbone length (Fig. 4 A), head diameter (Fig. 4 B), and volume (Fig. 4 C) revealed a selective population shift in dendritic spine morphology in layers II-III pyramidal neurons of the mPFC. A population shift towards longer dendritic spines was observed in HIV-1 Tg animals relative to control animals following MPH exposure [*χ* ^2^(12) = 99.1, *p* ≤ 0.001]. Assessment of dendritic spine head diameter revealed a population shift towards decreased head diameter in HIV-1 Tg animals relative to control animals [*χ* ^2^(10) = 25.3, *p* ≤ 0.005]. Measurement of dendritic spine volume revealed no significant alterations in dendritic spine morphology in HIV-1 Tg animals relative to controls [*χ* ^2^(15) = 14.9, p > 0.05].

**Figure 5:**
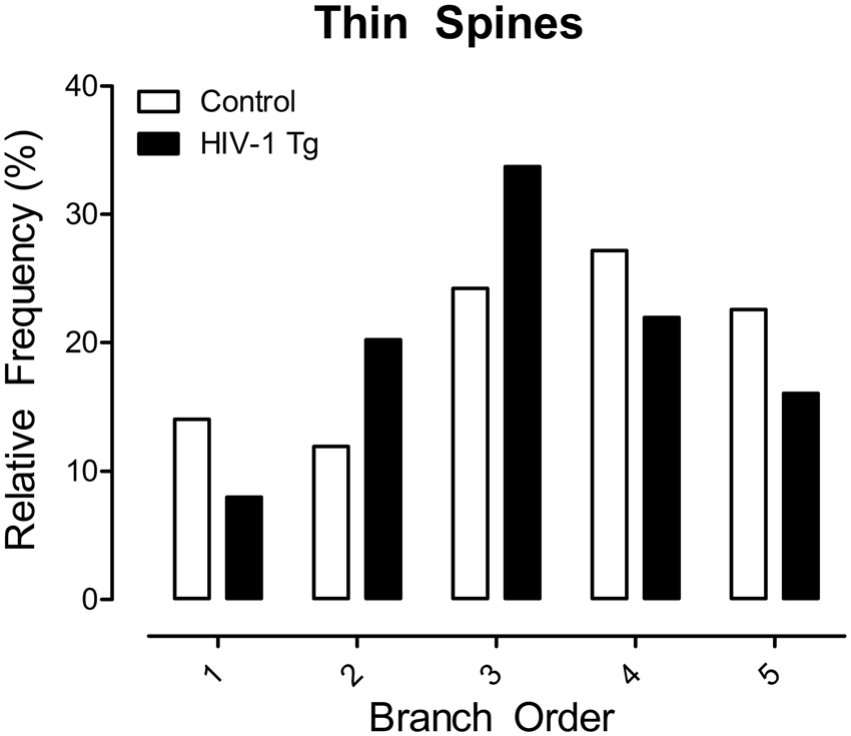
A histogram representing the distribution of thin spines on neuronal branches following repeated methylphenidate exposure is presented as a function of genotype (HIV-1 Tg vs. Control). HIV-1 Tg animals exhibited a population shift towards an increased relative frequency of thin spines on lower order branches relative to control animals.

Given the preponderance of thin spines in both HIV-1 Tg and control animals, a branch order analysis was subsequently conducted (Fig. 5). A significant shift towards an increased relative frequency of thin spines on lower order branches was observed in HIV-1 Tg animals relative to control animals following OSA of MPH [*χ* ^2^(4) = 708.7, *p* ≤ 0.001]. Thus, HIV-1 Tg animals exhibited a population shift towards longer spines with a decreased head diameter on lower order branches following repeated OSA of MPH; a shift that was far more robust than in the control animals.

### Examination of Individual Differences in HIV-1 Tg Animals

#### Approximately 42% of HIV-1 Tg animals displayed an improvement in temporal processing following OSA of MPH

**Figure 6:**
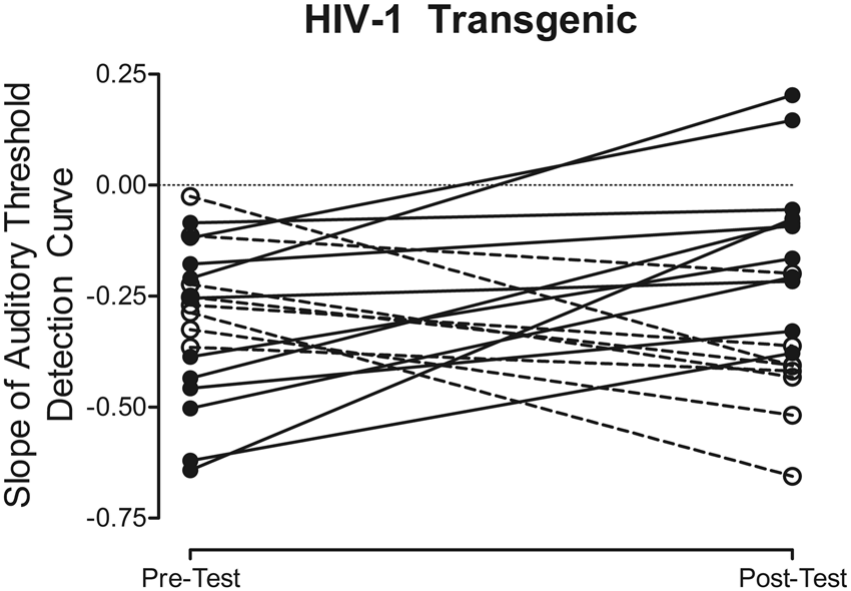
The slope of the auditory gap threshold detection curve for HIV-1 Tg animals is shown at the pretest and following the final day of oral self-administration (OSA) of methylphenidate (MPH; posttest). Nearly half (42%) of HIV-1 Tg animals exhibited an improvement in temporal processing following OSA of MPH, evidenced by a steeper auditory threshold detection curve at the posttest assessment.

Assessment of each HIV-1 Tg animal’s auditory gap threshold detection curve slope at the pretest and posttest revealed substantial between-subject variability (Fig. 6). Although many HIV-1 Tg animals displayed a relatively flatter auditory threshold detection slope following OSA of MPH, a number of animals displayed a steeper slope, suggesting an improvement in temporal processing. Specifically, approximately 42% (i.e., 8 out of 19) of HIV-1 Tg animals displayed improvements in temporal processing following OSA of MPH, evidenced by a positive change score.

#### Dendritic spine morphology in layers II-III pyramidal neurons of the medial prefrontal cortex was associated with temporal processing impairment in HIV-1 Tg animals

**Figure 7:**
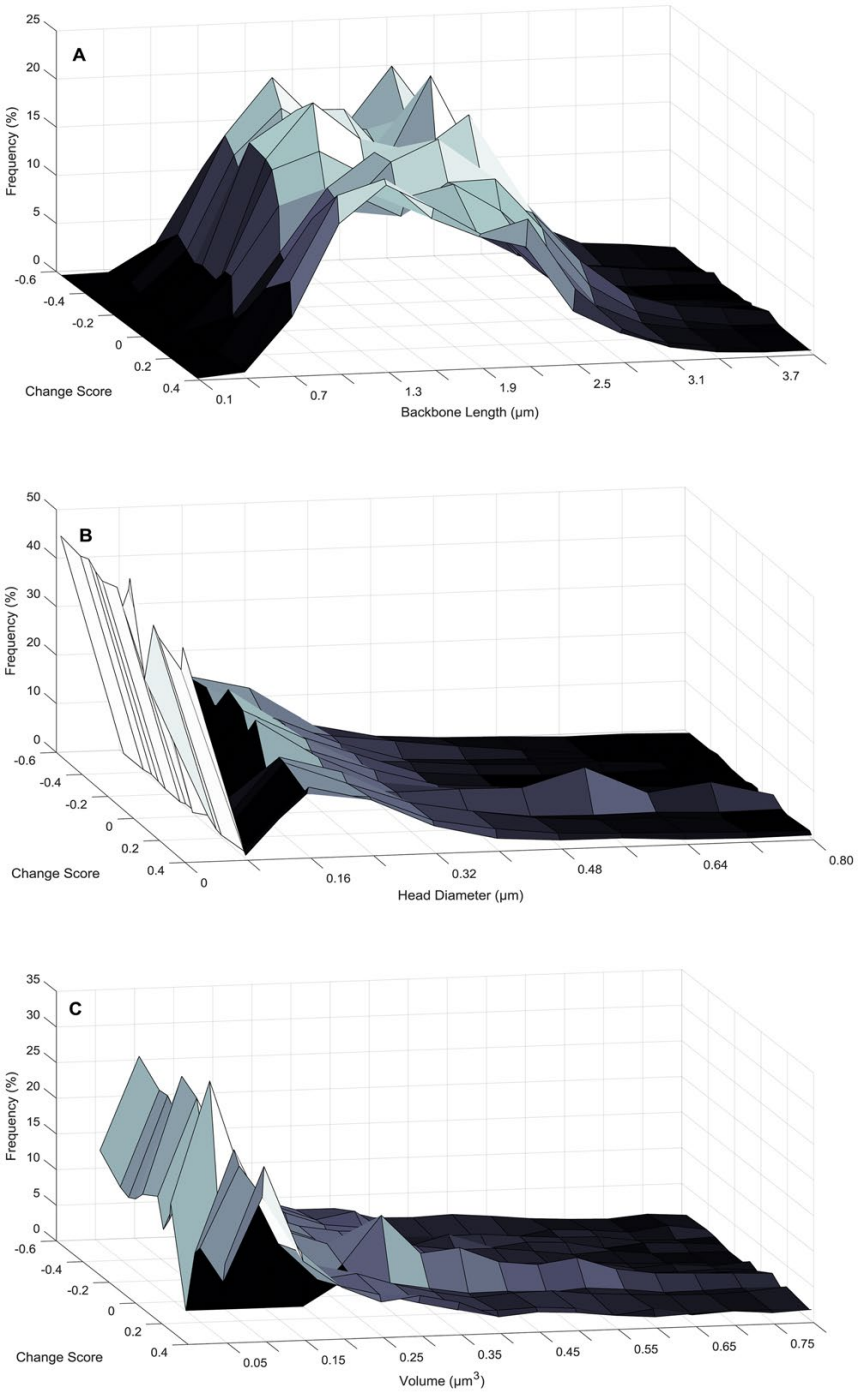
Three-dimensional surface plots representing the distribution of dendritic spine backbone length (**A**), head diameter (**B**), and volume (**C**) following repeated methylphenidate exposure are illustrated in HIV-1 Tg animals as a function of change score. The more positive the change score, the greater the population shift towards shorter spines (**A**) with increased head diameter (**B**) and increased volume (**C**); a shift similar to control animals.

Dendritic spine parameters, including backbone length (Fig. 7 A), head diameter (Fig. 7 B), and volume (Fig. 7 C), were subsequently examined in HIV-1 Tg animals dependent upon the magnitude and valence of the change score.

For the statistical analysis, animals were binned into 20^th^ percentile groupings based on their change score. The more positive the change score, the greater the population shift towards shorter dendritic spines [*χ* ^2^(48) = 339.9, *p* ≤ 0.001]. Measurement of dendritic spine head diameter revealed a population shift towards increased head diameter as the change score became more positive [*χ* ^2^(32) = 253.0, *p* ≤ 0.001]. An assessment of dendritic spine volume revealed that the more positive the change score, the greater a population shift towards increased volume [*χ* ^2^(60) = 190.1, *p* ≤ 0.001].

**Figure 8:**
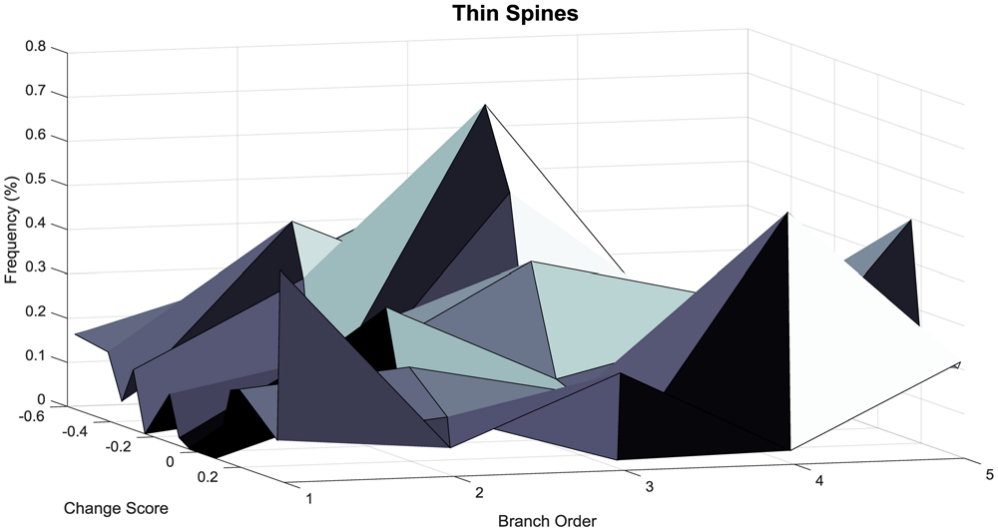
A three-dimensional surface plot representing the distribution of thin spines on neuronal branches following repeated methylphenidate exposure is presented in HIV-1 Tg animals as a function of change score. Positive change scores, indicating an improvement in temporal processing, were associated with an increased relative frequency of thin spines on higher order branches.

A branch order analysis was subsequently conducted on spines classified by Neurolucida 360 as thin spines (Fig. 8). The more positive the change score, the greater the population shift towards an increased relative frequency of thin spines on higher order branches [*χ* ^2^(16) = 2247.9, *p* ≤ 0.001]. Thus, an assessment of dendritic spine morphology dependent upon an improvement in temporal processing revealed that as the change score became more positive, a population shift towards shorter spines with increased head diameter and increased volume on higher order branches was observed; a shift similar to control animals.

#### Examination of the brain/behavior relationship provides a method for examining variability on a continuous scale

**Figure 9:**
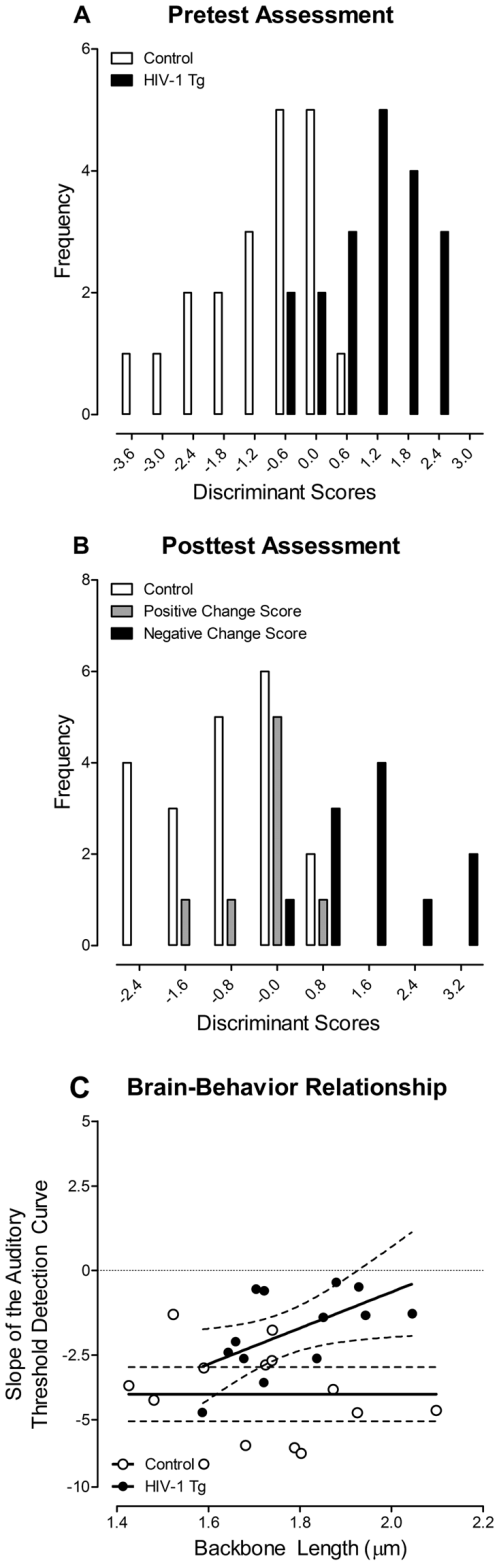
The effect of arbitrary classification, and thus loss of natural heterogeneity, was examined using a discriminant function analysis (DFA) and brain/behavior relationship. (**A**) The ability of gap threshold detection to classify HIV-1 Tg and control animals is illustrated as a function of the canonical variable (canonical correlation 0.74) and correctly identified (jackknife classification) group membership with 87.2% accuracy. (**B**) However, when HIV-1 Tg animals were arbitrarily classified into two groups (Positive Change Score (i.e., improvement in temporal processing) vs. Negative Change Score (i.e., temporal processing impairment)), dramatic reductions (i.e., 69.2% accuracy) in accurately identifying group members (jackknife classification) were observed. (**C**) A brain-behavior relationship between the slope (±95% Confidence Interval) of the auditory threshold detection curve was observed in HIV-1 Tg animals, but not control animals. In HIV-1 Tg animals, a linear increased in backbone length (µm) was observed as the slope of the auditory threshold detection curve approached 0.

At the pretest assessment, the  diagnostic utility of gap threshold detection was examined using a DFA to determine whether slope of the auditory gap threshold detection curve could accurately identify the presence of the HIV-1 transgene (Fig. 9 A). A DFA based on one variable (Auditory Gap Threshold Detection Slope) was used to separate the HIV-1 Tg and control animals (canonical correlation of 0.74). Presence of the HIV-1 transgene (jack-knifed classification) was diagnosed with 87.2% accuracy (Approximation of Wilks’ λ of 0.45, χ^2^(1) = 29.4, *p* ≤ 0.001).

At the posttest assessment, a DFA based on one variable (Auditory Gap Threshold Detection Slope) was used to separate the Positive Change Score, Negative Change Score, and control animals (Fig. 9 B; canonical correlation of 0.75). Animals were diagnosed, based on their impairment with 69.2% accuracy (Approximation of Wilks’ λ of 0.44, χ^2^(2) = 29.7, *p* ≤ 0.001). Thus, after arbitrary classification, which eliminates the variability observed in HIV-1 Tg animals, the diagnostic utility is dramatically reduced.

A brain/behavior relationship (Fig. 9 C) was observed between the slope of the auditory gap threshold detection curve and mean backbone length (µm) in HIV-1 Tg animals [β = 0.766, *r*(13) = 0.566, *p* ≤ 0.05), but not in control animals [*r*(14) = −0.228, *p* > 0.05]. HIV-1 Tg animals exhibited a linear increase in backbone length (µm) as the slope of the auditory threshold detection curve approached zero. Thus, use of a brain/behavior relationship allowed for the examination of variables on a continuous scale, without categorical classification, providing significant information that is otherwise unavailable.

## Discussion

NCI in the HIV-1 Tg rat are naturally heterogeneous, resembling the variability commonly observed in HIV-1 seropositive individuals. In the present study, OSA of MPH was used as a vehicle to a capture the natural heterogeneity in the HIV-1 Tg rat. Examination of the effect of genotype suggested that MPH failed to mitigate temporal processing deficits, assessed using gap threshold detection in a pretest-posttest experimental design, in the population of HIV-1 Tg animals sampled. However, an examination of the natural heterogeneity in HIV-1 Tg animals revealed that nearly half (i.e., 42%) of HIV-1 Tg animals exhibited an improvement in temporal processing following OSA of MPH immediately preceding the posttest assessment; an improvement that was associated with a population shift in dendritic spine morphology similar to control animals. In HIV-1 Tg animals, an increase in backbone length (µm) was associated with temporal processing impairment; a brain/behavior relationship not observed in control animals. Assessing the therapeutic efficacy of MPH revealed heterogeneity in the neural mechanisms underlying neurocognitive impairments, providing a key target for individualized therapeutic and diagnostic approaches for HAND.

HIV-1 Tg animals exhibited profound deficits in temporal processing which were not ameliorated by OSA of MPH. During the pretest assessment, both HIV-1 Tg and control animals exhibited a linear decrease in ASR as gap duration increased. However, HIV-1 Tg animals displayed a relative insensitivity to the manipulation of gap duration, evidenced by a flatter auditory gap threshold detection function, compared to control animals; observations which were consistent with previously reported deficits in auditory gap threshold detection^41^. Immediately preceding the posttest assessment, HIV-1 Tg and control animals completed their final OSA of MPH. Examination of the population of HIV-1 Tg animals sampled suggested that OSA of MPH failed to alter the auditory gap threshold detection curve. In contrast, OSA of MPH altered the auditory gap threshold detection function in control animals, evidenced by a relatively flatter gap duration function at the posttest assessment relative to the pretest assessment.

The effect of repeated MPH exposure on temporal processing deficits in the HIV-1 Tg rat (data not shown), investigated in cross-modal PPI (auditory, visual) and auditory gap-PPI, was consistent with observations in auditory gap threshold detection. HIV-1 Tg animals exhibited a relative insensitivity to the manipulation of ISI in cross-modal PPI and auditory gap-PPI at the pretest assessment^45^; an alteration in temporal processing which was not ameliorated by repeated MPH exposure. Control animals, however, did display significant alterations in cross-modal PPI and gap-PPI following repeated MPH exposure. Specifically, control animals exhibited a relative insensitivity to the manipulation of ISI, evidenced by a significantly flatter ISI function, in cross-modal PPI and gap-PPI at the posttest assessment relative to the pretest assessment^45^. Thus, at the genotype level, exposure to MPH failed to ameliorate temporal processing deficits in the population of HIV-1 Tg rats sampled.

Pyramidal neurons, characterized by one large apical dendrite and several shorter basal dendrites, are found primarily in brain structures associated with higher cognitive function, including the cerebral cortex^55^. Examination of pyramidal neurons from layers II-III of the mPFC following repeated MPH exposure revealed significant alterations in dendritic spine morphology. A population shift towards longer spines with decreased head diameter was observed in HIV-1 Tg animals; a shift which was not as robust in the population of control animals. Both HIV-1 Tg and control animals had an abundance of thin spines, characterized by a relatively thin, long neck and small head^56^. A population shift, with an increased relative frequency of thin spines on lower order branches, was observed in HIV-1 Tg animals relative to controls.

A closer examination of the natural heterogeneity in HIV-1 Tg animals revealed that approximately 42% of HIV-1 Tg animals exhibited an improvement in temporal processing following OSA of MPH. The significant individual variability in the therapeutic efficacy of MPH for the treatment of HAND resembles observations in patients with ADHD, where approximately 10–30% of patients fail to adequately respond to treatment^30^. The magnitude and valence of the change score was associated with a population shift in dendritic spine morphology. Specifically, the more positive the change score, the greater the population shift towards shorter dendritic spines with increased head diameter and increased volume on higher order branches; a shift similar to control animals. Contrary to reports by Hinkin et al^32^. in a predominantly male sample of HIV-1 seropositive adults (*n* = *16;* male, *n* = 15; female, *n* = 1), an improvement in temporal processing was not related to the severity of NCI at the pretest assessment. Determining the factors underlying individual differences in the therapeutic efficacy of MPH may provide for more individualized, and thus effective, treatments for HAND.

Improvements in temporal processing observed in HIV-1 Tg rats following OSA of MPH may be due, in part, to the blockade of both the neuronal DAT and NET^22,23,25^. A dose-dependent blockade of DAT has been observed^57^, with therapeutic doses of MPH (0.3–0.6 mg/kg) blocking more than 50% of DAT^23^. Notably, individual variability in responsiveness to MPH treatment may be explained by differences in DA activity, with low levels of DA cell activity leading to nonresponse^58^. The NET, which primarily clears extracellular DA in the PFC^59^, may also play a critical role in the mechanism of action for MPH^60^. In rats, co-administration of idazoxan, an α2 adrenoceptor antagonist, or SCH23390, a DA D1 receptor antagonist, with oral MPH reversed the cognitive-enhancing effects of MPH on a task dependent upon the PFC [i.e., delayed alternation;^61^]. Additionally, *in vivo* microdialysis revealed that DA and NE neurotransmission are preferentially activated in the PFC by low doses of MPH^25^. Thus, the improvement observed in temporal processing in a subset of HIV-1 Tg rats following OSA of MPH may be due, at least in part, to the blockade of both the DAT and NET, resulting in increased levels of extracellular DA.

Dendritic spines are sites of synaptic contact that serve as the main postsynaptic compartments of excitatory synapses^62^. Structural and morphological features of dendritic spines may reflect functionality and capacity for structural change^63^. Observations of a population shift towards decreased head diameter in HIV-1 Tg animals, relative to controls, may be indicative of decreased synaptic efficacy^64^, including DA neurotransmission^65^. DA receptors, which are concentrated in pyramidal neurons, are associated with postsynaptic density and may influence synaptic transmission, specifically at non-active synapses, subsequently mediating neurocognition^66^. Changes in dendritic spine morphology following treatment with other psychostimulants (e.g., cocaine, amphetamine) has been well-characterized in pyramidal neurons in the PFC [e.g.^67^] and medium spiny neurons in the nucleus accumbens [e.g.^49^]. Specifically, in pyramidal neurons, repeated exposure to either amphetamine or cocaine produced long-lasting structural modifications [e.g., increased number of dendritic branches, increased dendritic spine density;^67^]. Thus, neurocognitive changes resulting from treatment with MPH may result from changes in the levels of extracellular DA leading to alterations in synaptic connectivity in the PFC.

Diagnostic criteria commonly used for HAND (e.g., clinical ratings, deficits score, HAND classification system/Frascati criteria) categorically classify HIV-1 seropositive individuals, obfuscating the well-acknowledged natural variability observed across HIV-1 seropositive individuals^37^. In the present study, a DFA was used to statistically evaluate the effect of categorical classification on diagnosis in HIV-1 Tg animals. At the pretest assessment, a DFA revealed that one measure (i.e., the slope of the gap threshold detection curve) identified the presence of the HIV-1 transgene with 87.2% accuracy; results which are consistent with multiple studies suggesting the potential utility of PPI as a diagnostic screening tool for HAND^41,45^. At the posttest assessment, however, HIV-1 Tg animals were reclassified based on the magnitude and valence of their change score into two groups: Positive Change Score (i.e., improvement in temporal processing) and Negative Change Score (i.e., temporal processing impairment). At the posttest assessment, a DFA revealed that one measure (i.e., slope of the gap threshold detection curve) correctly classified only 69.2% of the animals. The arbitrary classification of HIV-1 Tg animals on one cognitive domain revealed a dramatic increase in misdiagnosis. Thus, there is a critical need for a method that can more objectively differentiate levels of impairment across the broad spectrum of HAND^10^.

A differential brain/behavior relationship was observed in HIV-1 Tg and control animals. HIV-1 Tg animals exhibited a linear increase in dendritic spine backbone length as the slope of the auditory gap threshold detection curve approached zero. Control animals, however, failed to display a significant brain/behavior relationship, evidenced by a horizontal fit. To our knowledge, the present study is the first to directly correlate dendritic spine parameters with NCI in HIV-1 Tg animals. Given the relationship between gap threshold detection and postmortem neuroanatomical measures, PPI may be a method for differentiating between levels of impairment in HAND, providing a novel diagnostic screening tool. The potential utility of PPI as an innovative diagnostic screening tool should be critically tested by examining whether early deficits in PPI are predictive of later neurocognitive deficits in higher order cognitive processes, including executive function.

Examination of the natural heterogeneity of HIV-1 Tg animals is one of the undeniable strengths of the statistical analyses in the present study. As illustrated by the DFA conducted following an arbitrary classification into two groups (i.e., Positive Change Score vs. Negative Change Score), dichotomization decreases variability and the diagnostic utility of auditory gap threshold detection. Dichotomization occurs when a continuous variable is split to create a binary variable (e.g., high vs. low), which is subsequently used in the statistical analysis as a between-groups factor^68^. The use of median-split designs, while common in the literature, has inherent limitations, including decreased power, inefficient effect size estimates, and loss of individual differences^69^. In sharp contrast, the pretest and posttest slope of the auditory gap threshold detection curve was used to calculate a continuous variable (i.e., change score) based on the magnitude and valence of change following MPH exposure; a variable which was subsequently used as the basis for dendritic spine analyses in HIV-1 Tg animals. Preserving individual differences provided a strong foundation for the assessment of a brain/behavior relationship in the HIV-1 Tg rat.

Despite the aforementioned strengths of the present study, a few caveats must also be identified. First, all animals, regardless of genotype, orally self-administered MPH during the pretest-posttest interval. Given the lack of saline control groups, the role of MPH in dendritic spine morphology is largely inferred via statistical approaches. Second, dendritic spine morphology was assessed across a variable timeframe (i.e., 3 months) after the final OSA of MPH due to natural time constraints present following DiOlistic labeling. However, variability in the time of sacrifice was experimentally controlled for by using a balanced design across time.

The HIV-1 Tg rat, originally developed by Reid *et al*.^70^, contains a *gag-pol-*deleted HIV-1 provirus with the transgene limited to chromosome 9. HIV-1 Tg animals, similar to HIV-1 seropositive individuals on cART, express viral proteins constitutively throughout development^71^. Although the HIV-1 Tg rat cannot be used to examine the effects of viral replication, it has been promoted as a valuable tool to model NCI commonly observed in HIV-1 [review,^72^]. No significant health disparities were observed in HIV-1 Tg animals assessed in the present study, consistent with previous reports^8,9,46^. Specifically, neither sensory system (i.e., visual or auditory), nor gross motoric impairments were observed in HIV-1 Tg rats through sixteen months of age^9^. To our knowledge, the present study is the first to examine the natural heterogeneity in the HIV-1 Tg rat, providing additional evidence for the translational relevance of the HIV-1 Tg rat for modeling HAND.

Collectively, these results provide strong evidence for significant heterogeneity in the HIV-1 Tg rat, resembling the variability observed within the population of HIV-1 seropositive individuals. MPH may provide an efficacious, adjunctive therapeutic for a subset of HIV-1 seropositive individuals. The use of auditory gap threshold detection as a novel diagnostic screening tool may provide an opportunity to differentiate between levels of impairment in HAND during the post-cART era. Alterations in dendritic spine morphology may underlie some of the cognitive impairments observed in HAND, providing a key target for the development of individualized therapeutic and diagnostic approaches for HAND.
